# Carnosol Increases Skeletal Muscle Cell Glucose Uptake via AMPK-Dependent GLUT4 Glucose Transporter Translocation

**DOI:** 10.3390/ijms19051321

**Published:** 2018-04-29

**Authors:** Filip Vlavcheski, David Baron, Ioannis A. Vlachogiannis, Rebecca E. K. MacPherson, Evangelia Tsiani

**Affiliations:** 1 Department of Health Sciences, Brock University, St. Catharines, ON L2S 3A1, Canada; fv11vi@brocku.ca (F.V.); db15qv@brocku.ca (D.B.); yv17av@brocku.ca (I.A.V.); rmacpherson@brocku.ca (R.E.K.M.); 2 Centre for Bone and Muscle Health, Brock University, St. Catharines, ON L2S 3A1, Canada

**Keywords:** carnosol, glucose uptake, muscle, AMPK

## Abstract

Skeletal muscle is a major insulin-target tissue and plays an important role in glucose homeostasis. Insulin action in muscle activates the phosphatidylinositol-3 kinase (PI3K)/Akt signaling pathway causing the translocation of intracellularly stored GLUT4 glucose transporters to the plasma membrane and increased glucose uptake. Impaired insulin action in muscle results in insulin resistance and type 2 diabetes mellitus (T2DM). Activation of the energy sensor AMP-activated kinase (AMPK) increases muscle glucose uptake and the use of AMPK activators is viewed as an effective strategy to combat insulin resistance. Rosemary extract (RE) has been shown to stimulate muscle AMPK and glucose uptake, but the exact components responsible for these effects are unknown. In the current study, we investigated the effect of carnosol, a RE polyphenol, in L6 rat muscle cells. Carnosol stimulated glucose uptake in L6 myotubes in a dose- and time-dependent manner, did not affect Akt, increased AMPK phosphorylation and plasma membrane GLUT4 levels. The carnosol-stimulated glucose uptake and GLUT4 translocation was significantly reduced by the AMPK inhibitor compound C (CC). Our study is the first to show an AMPK-dependent increase in muscle glucose uptake by carnosol. Carnosol has potential as a glucose homeostasis regulating agent and deserves further study.

## 1. Introduction

Skeletal muscle tissue is a primary insulin target that accounts for approximately 80% of insulin-mediated glucose uptake in the postprandial state and plays a major role in glucose homeostasis [1,2]. The binding of insulin to its receptor increases the receptor’s tyrosine kinase activity, inducing the downstream activation of phosphatidylinositol-3 kinase (PI3-K) and serine/threonine kinase Akt/PKB and GLUT4 glucose transporter translocation from an intracellular storage site to the plasma membrane, allowing glucose entry into muscle cells [1,3]. The activation of PI3-K by insulin leads to the phosphorylation of the 3 position hydroxyl group of the inositol ring of phosphatidylinositol, generating phosphatidylinositol (3,4,5)-triphosphate (PIP_3_) and downstream activation of phosphoinositide-dependent kinases (PDKs) and Akt activation [1,3,4]. The PI3K/Akt signaling pathway plays a key role in insulin-stimulated GLUT4 glucose transporter translocation [1,3,4,5,6] and impairments in this cascade lead to reduced insulin-induced glucose transport into muscle cells contributing to insulin resistance and type 2 diabetes mellitus (T2DM) [1,4,7].

The AMP-activated protein kinase (AMPK) integrates nutritional and hormonal signals and regulates cell metabolism. It is a serine/threonine kinase that acts as a cellular energy sensor; it consists of three subunits: a catalytic (α) and two regulatory (β and γ) subunits [8,9,10,11]. An increase in the cellular AMP/ATP ratio leads to the activation of AMPK, resulting in the inhibition of ATP consuming biosynthetic pathways and the activation of ATP generating pathways [8,9,10,11]. The activation of AMPK increases insulin sensitivity, glucose tolerance, and physical endurance [8]. AMPK is activated by exercise/contraction [12], metformin [13,14], and many polyphenols such as naringenin [15] and resveratrol [16]. Skeletal muscle glucose uptake through the stimulation of AMPK is considered a targeted approach to control blood glucose homeostasis.

Carnosol is a phenolic terpene (diterpene) found in rosemary and reported to have anti-inflammatory, antioxidant, and anticancer effects [17,18,19,20]. More recently, antihyperglycemic and anti-diabetic effects of rosemary and rosemary polyphenols have been reported [21,22,23,24].

Carnosol was found to inhibit pancreatic lipase activity (half maximal inhibitory concentration IC_50_ value: 4.4 μg/mL (13 μM)) [25]. In another in vitro study, carnosol was found to activate the human peroxisome proliferator-activated receptor γ (PPARγ) [26], the major target of the antidiabetic glitazone type of drugs. The activation of PPARγ leads to reduced blood glucose and fatty acid levels. In HepG2 hepatocytes, carnosol (20–40 μM) was found to significantly reduce the intracellular triglyceride synthesis that was associated with significant inhibition of diacylglycerol acyltransferase (DGAT) [27], the enzyme that catalyzes the formation of triglycerides from acetyl-CoA and diacylglycerol, and its inhibition has been proposed as a target approach for T2DM and obesity treatment. In 3T3-L1 adipocytes, carnosol (3 μM) inhibited the differentiation of pre-adipocytes into mature adipocytes and increased the intracellular levels of the antioxidant glutathione (GSH) [28].

Administration of carnosol (1, 5, 10 mg/kg/day) in streptozotocin-induced diabetic rats resulted in reduced blood glucose, triglyceride, total cholesterol, and low density lipoprotein (LDL) levels in the treated group compared to untreated group [29]. These effects were associated with reduced plasma levels of markers of inflammation and reduced oxidative stress (IL-6, TNFα, MDA, GST, SOD, CAT) [29].

In recent studies, we found direct effects of rosemary extract [21], as well as the rosemary polyphenols carnosic acid [22] and rosmarinic acid [23], on skeletal muscle cells whereby glucose uptake and phosphorylation/activation of AMPK were increased. The limited in vitro and in vivo studies mentioned above indicate a potential of carnosol to regulate blood glucose levels. However, the mechanisms implicated in the antihyperglycemic effects of carnosol are not known. In the present study, we examined the direct effects of carnosol on muscle cell glucose uptake and investigated the mechanisms involved. The effect of carnosol in muscle cells and its mechanism of action have never been investigated before, making this study the first of its kind. The insights into the bioactive properties of carnosol and the mechanism of action may significantly contribute toward enhancing the understanding of its properties, and this improved understanding may ultimately be utilized further in order to prevent/treat insulin resistance and T2DM. The consumption of carnosol has been approved by the European Union (EU) and given a Generally Recognized as Safe (GRAS) status by the United States Food and Drug Administration (FDA) [18]. This, together with the limited evidence of antihyperglycemic effects of carnosol, prompted us to investigate its direct effects and action in muscle cells.

Carnosol increased glucose uptake in L6 myotubes to levels similar to those seen with insulin and the anti-diabetic drug metformin. Carnosol increased AMPK phosphorylation/activation although it did not affect Akt phosphorylation/activation. GLUT4 glucose transporter translocation to plasma membrane was increased by carnosol treatment. Compound C, an AMPK inhibitor, significantly reduced the carnosol-stimulated glucose uptake and GLUT4 translocation, thereby indicating the involvement of AMPK. Carnosol appears to be a strong activator of AMPK and further studies are required to examine its potential use against insulin resistance and T2DM.

## 2. Results

**Figure 1:**
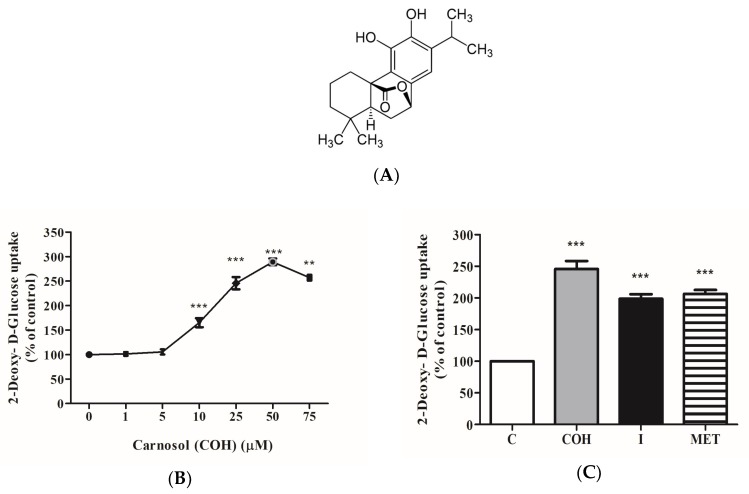
(**A**) Structure of Carnosol (C_20_H_26_O_4_) (**B**,**C**) Effects of carnosol on glucose uptake. (**B**) Dose response: Serum deprived L6 myotubes were incubated without (0 μM) or with the indicated concentrations of carnosol. (**C**) Myotubes were incubated without (control, C) or with 25 µM carnosol (COH) (4 h), 100 nM insulin (I) (0.5 h), or 2 mM metformin (MET) (2 h) followed by 2-deoxy-d-glucose uptake measurements. Results are the mean ± standard error (SE) of three to six independent experiments performed in triplicate. ** *p* < 0.01, *** *p* < 0.001, vs. control.

The structure of carnosol (C_20_H_26_O_4_) is shown in Figure 1A. Exposure of L6 myotubes to different concentrations of carnosol (1, 5, 10, 25, 50, 75 μM) for 4 h resulted in a dose-dependent increase in glucose uptake. A significant increase was seen with 10 μM of carnosol (165.1 ± 9.1% of control) and maximum response was seen with 50 μM (303 ± 14.5% of control) (Figure 1B). Importantly, the response of the cells to 25 μM carnosol (245 ± 12.6% of control) was comparable to maximum insulin and metformin response (193.4 ± 8.1%, 206 ± 6.3% of control, respectively) (Figure 1C).

**Figure 2:**
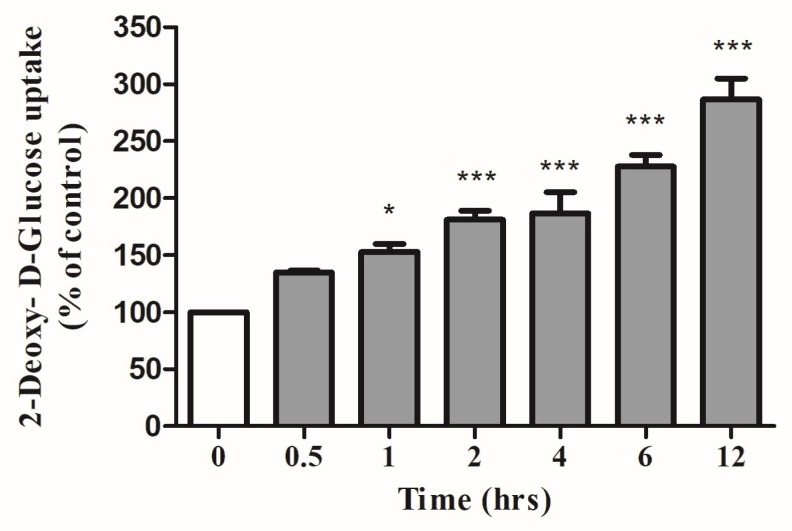
Effects of carnosol on glucose uptake. Time-course: Serum deprived L6 myotubes were incubated without (0) or with 25 µM carnosol for the indicated time followed by 2-deoxy-d-glucose uptake measurements. Results are the mean ± SE of three to four independent experiments performed in triplicate. * *p* < 0.05, *** *p* < 0.001, vs. control (0 h).

Next, we examined the time-course of the action of carnosol. The cells were exposed to 25 μM of carnosol for different periods of time, ranging from 30 min to 24 h. A non-significant increase (134 ± 1.77% of control) was seen after 0.5 h of exposure. A significant increase (153 ± 6.8% of control) was seen after 1 h of exposure to carnosol (Figure 2). Exposure to carnosol for 2 or 4 h increased glucose uptake to 181 ± 7.8% and 187 ± 18.5% of control, respectively, and a response of 286.5 ± 18.51% of control was seen after 12 h of exposure (Figure 2). Longer exposure (up to 24 h) resulted in a significant increase in glucose uptake (339.6 ± 19.5% of control).

**Figure 3:**
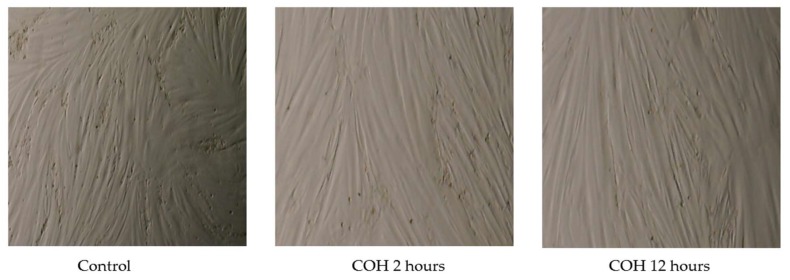
Effect of carnosol on L6 cell morphology. Cells were treated without (Control) or with 25 μM carnosol (COH) for 2 or 12 h. The cells were photographed using EVOS XL Core imaging system at magnification ×20.

To investigate any potential cell damaging effects of carnosol under these conditions, we examined cell morphology and cell viability. Our data (Figure 3) show no changes in cell morphology after treatment with carnosol for 2 or 12 h. Additionally, we used the trypan blue exclusion assay to assess the effects of carnosol on cell viability. Treatment with carnosol for 2 or 12 h did not affect cell viability (cell viability: carnosol 2 h: 99%, carnosol 12 h: 99.3% of control).

**Figure 4:**
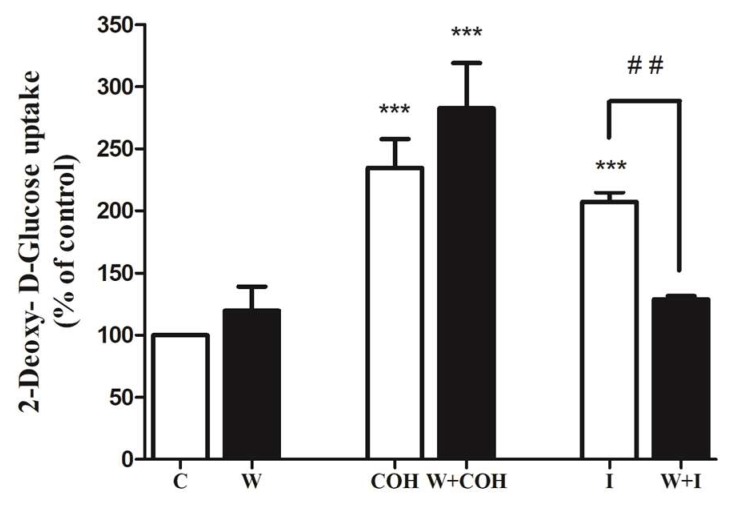
Carnosol-stimulated glucose uptake. Role of PI3K: Myotubes were serum deprived and incubated in the absence (control, C) or presence of 100 nM wortmannin (W) for 15 min followed by treatment with or without 25 µM carnosol (COH) for 4 h, or 100 nM insulin (I) for 0.5 h and 2-deoxy-d-glucose uptake measurements. Data are the mean ± SE of three to four experiments performed in triplicate. *** *p* < 0.001, vs. control (C), ## *p* < 0.01, vs. insulin (I).

Next, we investigated the signaling cascades involved and examined the PI3K-Akt cascade that is established to mediate the insulin-stimulated glucose uptake. We used the PI3K inhibitor wortmannin. Wortmannin did not affect the carnosol-stimulated glucose uptake (COH: 234 ± 22.99%, W+COH: 282 ± 36.50% of control), thus indicating that PI3K is not involved in the action of carnosol (Figure 4). Importantly, wortmannin significantly reduced the insulin-stimulated glucose uptake (I: 207 ± 7.8%, W+I: 128 ± 2.7% of control) (Figure 4).

**Figure 5:**
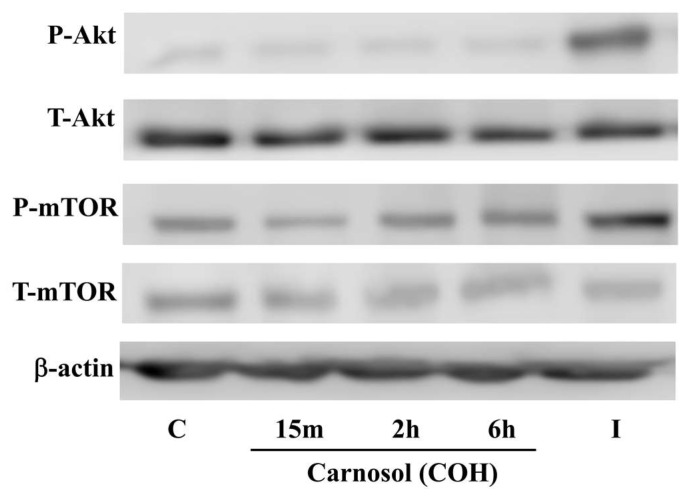
Effects of carnosol on Akt signaling cascade. Whole cell lysates from cells treated without (control, C) or with 25 µM carnosol (COH) (15 min, 2, 6 h), or 100 nM insulin (I) (15 min), were prepared, resolved by SDS-PAGE, and immunoblotted for total Akt, phospho-(Ser473) Akt, total mTOR, phospho-mTOR, or β-actin.

The activation of PI3K by insulin leads to downstream activation of Akt and therefore we examined Akt phosphorylation/activation and expression by Western blotting (Figure 5). Exposure of L6 myotubes to 25 μM of carnosol for 15 min, 2, or 6 h did not affect Akt phosphorylation/activation. On the other hand, exposure of the cells to insulin (100 nM, 15 min) resulted in a robust increase in Akt phosphorylation/activation. The total levels of Akt were not affected by any treatment (Figure 5). The activation of Akt by insulin leads to downstream activation of the mammalian target of rapamycin (mTOR) and therefore we examined mTOR phosphorylation as an indicator of Akt activation. Treatment with carnosol did not affect mTOR phosphorylation/activation, while treatment with insulin resulted in a significant increase in mTOR phosphorylation/activation (Figure 5). These data clearly indicate no effect of carnosol on the PI3K-Akt signaling cascade. The levels of total mTOR were not affected by any treatment. The levels of β-actin, used as loading control, also remained unchanged by cell treatment.

**Figure 6:**
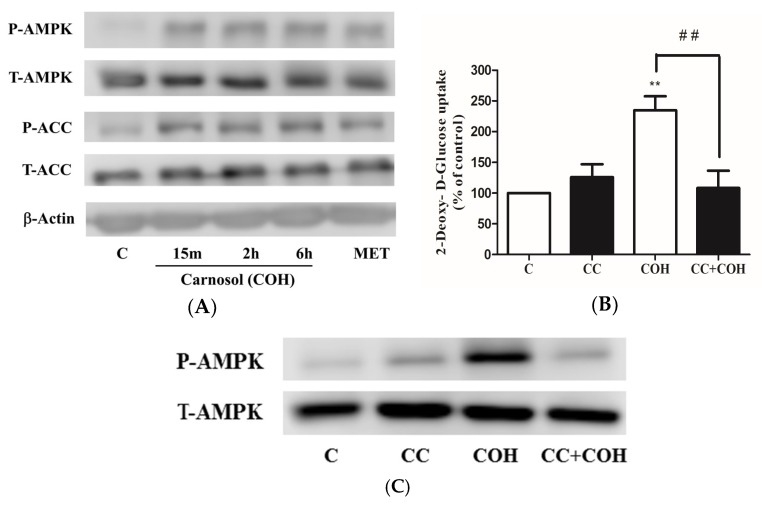
Effects of carnosol on AMP-activated protein kinase (AMPK) signaling cascade. (**A**) Whole cell lysates from cells treated without (control, C) or with 25 µM carnosol (15 min, 2, 6 h), or 2 mM metformin (MET) (2 h) were prepared, resolved by SDS-PAGE, and immunoblotted for phospho-AMPK, total AMPK, phospho-ACC, total ACC, or β-actin; (**B**) Cells were incubated in the absence (control, C) or the presence of 25 µM compound (C) (CC) for 0.5 h followed by exposure to 25 µM carnosol (COH) (2.5 h) and 2-deoxy-d-glucose uptake measurements. Data are the mean ± SE of three to four experiments performed in triplicate. ** *p* < 0.01, vs. control (C), ## *p* < 0.01, vs. carnosol (COH); (**C**) Whole cell lysates from cells treated without (control, C) or with 25 µM CC for 0.5 h followed by treatment with carnosol (2 h) were prepared, resolved by SDS-PAGE, and immunoblotted for phospho- or total AMPK.

Next, we examined the effect of carnosol on AMPK phosphorylation/activation. Exposure of the cells to 25 μM of carnosol resulted in a significant increase in AMPK phosphorylation/activation that was comparable to the effect of metformin (Figure 6A). Furthermore, the phosphorylation of acetyl-CoA carboxylase (ACC), the downstream target of AMPK that is established as an indicator of AMPK activation, was significantly increased by carnosol. The total levels of AMPK and ACC were not changed by any treatment (Figure 6A). We also examined the effect of the AMPK inhibitor compound C (CC) on the carnosol-stimulated glucose uptake. Exposure of the cells to CC abolished the carnosol-stimulated glucose uptake (COH: 234 ± 23%, CC+COH: 108 ± 28.11% of control) (Figure 6B), thus indicating an involvement of AMPK in this response. In addition, we investigated the effect of CC on AMPK. Treatment with CC abolished the COH-induced phosphorylation of AMPK, thus indicating that in the present study treatment with CC provided effective inhibition of AMPK phosphorylation/activation (Figure 6C).

**Figure 7:**
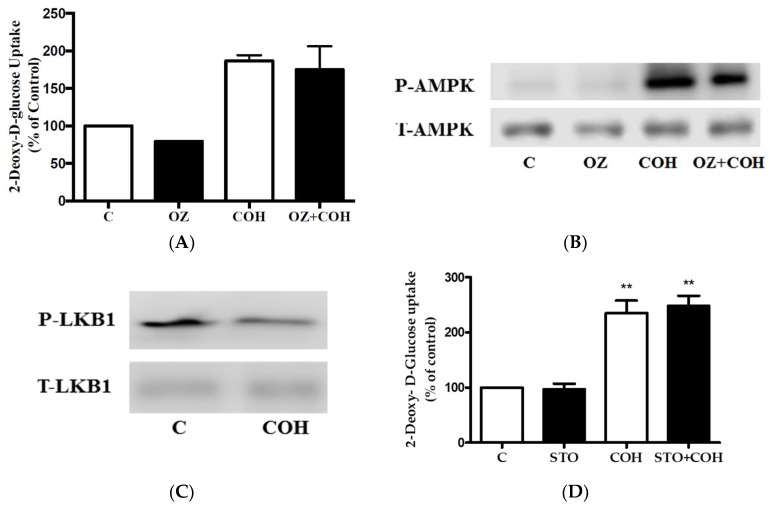
Role of transforming growth factor-β-activated kinase 1 (TAK1), liver kinase B1 (LKB1), and calcium/calmodulin-dependent protein kinase (CaMKK) in the COH-induced effects: (**A**,**D**) Myotubes were serum deprived and incubated in the absence (control, C) or presence of 2.5 µM (5*Z*)- oxozeaenol (OZ) (**A**) or 27 µM STO-609 (STO) (**D**) for 1 h followed by the addition of 25 µM carnosol (4 h) and 2-deoxy-d-glucose uptake measurements. Data are the mean ± SE of two experiments performed in triplicate; (**B**) Whole cell lysates from cells treated without (control, C) or with 2.5 µM (5*Z*)- oxozeaenol (OZ) 1 h followed by treatment without or with 25 µM carnosol (4 h), were prepared, resolved by SDS-PAGE, and immunoblotted for phospho- or total AMPK (**B**) or phospho- or total LKB1 (**C**). ** *p* < 0.01, vs. control (C).

In an attempt to delineate the mechanism of AMPK activation by carnosol, we investigated the involvement of the upstream regulators of AMPK, transforming growth factor-β-activated kinase 1 (TAK1) [10,30], liver kinase B1 (LKB1) [10], and calcium/calmodulin-dependent protein kinase (CaMKK) [10]. We investigated the involvement of TAK1 in the COH-stimulated glucose uptake by using (5*Z*)-oxozeaenol (OZ), a potent inhibitor of TAK1 [31]. Treatment with OZ did not affect the basal glucose uptake (Figure 7A). The COH-stimulated glucose uptake was not affected by OZ treatment, indicating that TAK1 is not involved in this response (Figure 7A). We also investigated the effect of OZ on AMPK. Treatment with OZ alone did not affect AMPK phosphorylation or expression (Figure 7B). Furthermore, OZ did not have an effect on the COH-induced AMPK phosphorylation (Figure 7B). The liver kinase B1 (LKB1) is another upstream regulator of AMPK [32]. Treatment with COH did not affect LKB1 phosphorylation/activation (Figure 7C). The activation of the CaMKK leads to downstream activation of AMPK and we investigated if CaMKK is involved in the carnosol-stimulated glucose uptake by using the CaMKK inhibitor STO-609. STO-609 did not affect the basal or the carnosol-induced glucose uptake (Figure 7D). Collectively, these data indicate that TAK1, LKB1, and CaMKK are not involved in the COH-induced effects.

**Figure 8:**
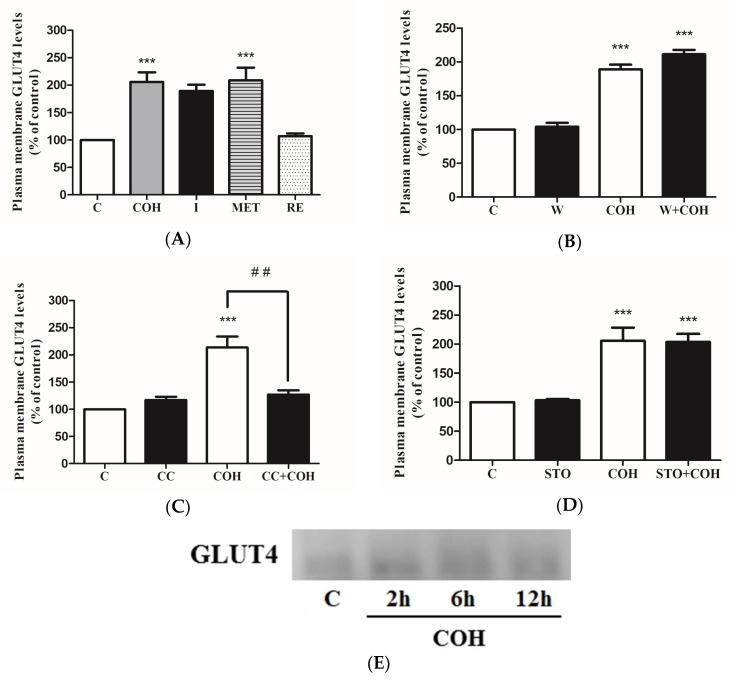
Effect of carnosol on GLUT4 glucose transporter. GLUT4myc overexpressing L6 myotubes were treated without (control, C) or with 25 µM carnosol) (4 h), 100 nM insulin (I) (0.5 h), 2 mM metformin (MET) (4 h), or 5 µg/mL rosemary extract (RE) (4 h) (**A**), 100 nM wortmannin (W) for 15 min (**B**), 25 µM compound C (CC) for 0.5 h (**C**), or 27 µM STO-609 (STO) for 1 h (**D**) followed by treatment without or with 25 µM carnosol (4 h). After treatment, GLUT4 transporter translocation measurements were performed. Results are mean ± SE of three to five independent experiments performed in triplicate and expressed as percentage of control, *** *p* < 0.001, ## *p* < 0.01. (**E**) Whole cell lysates from L6 parental myotubes treated without (control, C) or with 25 µM carnosol for 2, 6, and 12 h were prepared, resolved by SDS-PAGE, and immunoblotted for total GLUT4.

The insulin-induced glucose uptake in L6 cells is mainly mediated by GLUT4 glucose transporter translocation from an intracellular storage pool to plasma membrane. We examined whether carnosol induces GLUT4 translocation by measuring plasma membrane GLUT4 levels in GLUT4myc overexpressing L6 cells. Carnosol significantly increased plasma membrane GLUT4 levels (COH: 205 ± 17.56% of control) similar to insulin and metformin (189 ± 11.68%, 208 ± 22.80% of control, respectively), (Figure 8A). Treatment with rosemary extract (RE) did not increase GLUT4 to the plasma membrane (107 ± 5.20% of control). The carnosol-mediated GLUT4 glucose transporter translocation was not affected by the PI3K inhibitor, wortmannin (COH: 189 ± 6.80%, W+COH: 211 ± 6.50% of control) (Figure 8B), indicating that the PI3K-Akt cascade is not involved in the action of carnosol. Importantly, the carnosol-mediated GLUT4 glucose transporter translocation was significantly reduced by the AMPK inhibitor, compound C (COH: 214 ± 20.21%, CC+COH: 127 ± 7.86% of control) (Figure 8C), indicating that AMPK is involved. We also investigated whether CaMKK is involved in the carnosol-mediated GLUT4 glucose transporter translocation by using the CaMKK inhibitor STO-609. STO-609 did not affect the carnosol-induced increase in GLUT4 plasma membrane levels (COH: 206 ± 22.68%, STO+COH: 203 ± 14.05% of control), indicating no involvement of CaMKK (Figure 8D). In addition, we examined the effect of carnosol treatment on total GLUT4 levels. Treatment of L6 parental cells with 25 µM carnosol for 2, 6, or 12 h had no effect on the total GLUT4 levels (Figure 8E).

## 3. Discussion

Skeletal muscle is the primary tissue for insulin-stimulated glucose uptake, plays a paramount role in the regulation of blood glucose levels, and is therefore recognized as an important therapeutic target tissue for insulin resistance and T2DM. The present study is the first to report a significant increase in muscle cell glucose uptake by micromolar concentrations of carnosol. Although there are no other studies examining the biological effects of carnosol in muscle cells, two other studies exist examining the effects of carnosol in other insulin target tissues, namely adipocytes [28] and hepatocytes [27], and in agreement with our study, significant biological effects were seen with micromolar concentrations of carnosol. Carnosol at 10 μM inhibited 3T3L1 adipocyte differentiation [28], while in Hep G2 hepatocytes a significant inhibition of intracellular triglyceride synthesis was seen with 20–40 μM carnosol [27].

The increase in muscle glucose uptake by insulin is mainly due to the translocation of GLUT4 glucose transporters from an intracellular storage site to the plasma membrane [33,34,35,36,37], a response that can be seen acutely within 30 min. On the other hand, the time-course of the antidiabetic drug metformin on muscle glucose uptake is more prolonged than insulin. In our lab, exposure of the cells to metformin for a minimum of 2 h is required to see an effect [22,23], and these observations are in agreement with other studies [38]. Overall, the data from the present study, together with the data obtained in other studies, indicate that the time-course of carnosol action is closer to the time-course of metformin. It should be noted that longer exposure (up to 12–24 h) of muscle cells to insulin or metformin results in increased glucose uptake due to increased GLUT expression [33,34,35,36]. Based on the past insulin and metformin data [22,23,38] and on the data from the present study, we assumed that the acute (1–4 h) increase in glucose uptake by carnosol treatment is due to GLUT translocation, while the more prolonged (12–24 h) effect may be due to modulation of GLUT expression. We measured total GLUT4 levels in parental cells and found no changes in response to carnosol treatment.

The insulin-stimulated glucose uptake in skeletal muscle cells is mediated by the PI3K-Akt signaling pathway and inhibition of PI3K [4] or Akt [7] completely abolishes this response. The carnosol-mediated glucose uptake was not affected by wortmannin, a PI3K irreversible inhibitor [39,40], indicating that PI3K is not involved in the mechanism of action of carnosol. On the other hand, the insulin-stimulated glucose uptake was significantly reduced by wortmannin, thus providing strong evidence that in our study wortmannin was biologically active and effective in blocking PI3K activity. We measured phosphorylation of Akt on -Ser473, which correlates with kinase activity and is used as a marker of its activation [41,42], and we found no effect of carnosol, while insulin induced a marked increase in Akt phosphorylation/activation. Furthermore, carnosol did not affect the phosphorylation/activation of mTOR, the downstream target of Akt [3], in contrast to a robust increase seen with insulin. Together, these data clearly indicate that while the insulin-stimulated glucose uptake is dependent on the PI3K-Akt cascade, the carnosol-stimulated glucose uptake is independent of the PI3K-Akt cascade.

AMPK functions as an energy sensor and metabolic switch that phosphorylates downstream key target proteins involved in lipid metabolism, fatty acid oxidation, and glucose uptake. Overall, the activation of AMPK leads to switching off ATP consuming biosynthetic pathways and turning on ATP producing pathways such as fatty acid oxidation and glucose uptake. Carnosol increased the phosphorylation of AMPK on Thr172, which is highly correlated with increased AMPK kinase activity [9,10,43]. In addition, carnosol increased the phosphorylation of the downstream target of AMPK, acetyl-CoA carboxylase (ACC), which is routinely used as a proxy of AMPK activity in various studies [44]. These data clearly indicate that carnosol is a strong AMPK activator. Compound C is often used to show the involvement of AMPK in a specific response. It should be mentioned that compound C has a broad spectrum and may affect other protein kinases. Therefore, future experiments focusing on knockdown or knockout of AMPK should be performed in order to better address the role of AMPK in mediating the effects of carnosol. Our study is the first to report a robust phosphorylation/activation of AMPK in muscle cells. Although there are no other studies examining the effect of carnosol on AMPK in any other insulin-target tissue, our data are in agreement with a study in prostate cancer cells (PC3) that found an increase in AMPK activation and inhibition of its downstream effector mTOR/p70 S6K/4E-BP1 pathway by carnosol (10–70 µM) [45].

We measured plasma membrane GLUT4 levels in L6 cells overexpressing GLUT4. Our data showed that there was a significant increase in plasma membrane levels of GLUT4 with carnosol treatment. Importantly, this increase was comparable to the response seen with maximum insulin. We have investigated the effects of rosemary extract [21], carnosic acid [22], and rosmarinic acid [23] in L6 muscle cells in previous studies, but we did not observe any effect of these treatments on GLUT4 translocation. Treatment with rosemary extract (RE) did not increase plasma membrane GLUT4 levels in contrast to the robust increase seen with carnosol treatment (Figure 8A). These data suggest that carnosol at levels found in rosemary extract may not be sufficient to elicit a response and/or that other components present in rosemary extract may counteract the effect of carnosol. In addition, in our past investigation we did not see any effect on GLUT4 translocation by the polyphenols resveratrol [16] and naringenin [15]. In the present study, treatment of the cells with carnosol showed similar effects with that of metformin treatment in terms of glucose uptake stimulation (Figure 1C), AMPK activation (Figure 6A), and GLUT4 glucose transporter translocation (Figure 8A), all of which suggest a strong potential of carnosol to be used as metformin against insulin resistance and towards glucose homeostasis regulation. The effect of carnosol on GLUT4 translocation is very novel. Compound C and not wortmannin significantly inhibited the carnosol-mediated increase in plasma membrane GLUT4 levels, thus strongly indicating the involvement of AMPK. We also used STO-609, an established inhibitor of CaMKK [46], the kinase upstream of AMPK, in an attempt to further delineate the mechanism of carnosol action. STO-609 did not affect the carnosol-stimulated GLUT4 translocation, indicating that this kinase is not involved in the mechanism of action of carnosol. Overall, our data indicate no effect on total GLUT4 levels but a significant increase in GLUT4 translocation by carnosol treatment.

Currently, there are no studies investigating the mechanism of AMPK activation by carnosol. AMPK activation can occur by an increase in the AMP/ATP ratio as well as the activation of its upstream kinases TAK1 [10,30], LKB1, and CaMKK, [8,9,10,11]. Carnosol may allosterically modulate the activity of AMPK, increase the activity of its upstream kinases including TAK1, LKB1, and CaMKK [10], or lead to an increase in the AMP/ATP ratio as a result of the inhibition of the mitochondrial complex 1. The TAK inhibitor OZ did not affect the carnosol-induced AMPK phosphorylation (Figure 7B). In addition, we found that the CaMKK inhibitor STO did not affect the carnosol-induced AMPK phosphorylation, and LKB1 was not affected by carnosol treatment (Figure 7C). Together, these data suggest that the carnosol-induced AMPK T172 phosphorylation may be mediated by autophosphorylation and requires further study. In addition, more robust future experiments focusing on TAK1, LKB1, and CaMKK knockout are required to address the role of these kinases in carnosol-induced AMPK phosphorylation. It is interesting to consider, and it should be further elucidated, how the phosphorylation of AMPK at T172 is increased by carnosol treatment. Is it possible for it to be mediated by unknown upstream kinase(s) or, as mentioned above, by autophosphorylation? Generally, it is well-documented that T172 phosphorylation is highly dependent on these upstream kinases, especially LKB1 and CaMKK. For instance, glucose starvation, which depletes cellular energy level to activate AMPK in a LKB1-dependent manner in many cell lines, does not induce AMPK T172 phosphorylation in LKB1 negative HeLa cells [47]. In contrast, Ca^2+^ ionophore, which increases cellular Ca^2+^ levels to activate CaMKKb, does induce AMPK T172 phosphorylation in the same cell line [48]. Studies have demonstrated that metformin significantly inhibits the mitochondrial complex 1 [49,50], thereby activating AMPK. It is possible that carnosol may act in a manner similar to metformin and inhibit mitochondrial complex 1, resulting in AMPK activation? In the conditions where there are decreasing ATP and concomitantly increasing AMP, LKB1 plays important roles in AMPK activation [51]. It appears from our studies that LKB1 is not involved and therefore the use of carnosol may provide the opportunity to delineate a new mechanism of AMPK activation.

It is not known how carnosol mediates its effects, whether it binds to any receptors or transporters or whether it enters the cells. In bovine aortic endothelia cells, carnosol inhibited the H_2_O_2_-induced reactive oxygen species (ROS) levels by a mechanism involving the estrogen receptor [52]. Estrogen receptor (ERα and ERβ) antagonists abolished the antioxidant effects of carnosol. Carnosol acted as an estrogen receptor agonist, which may explain the cardio-protective properties of carnosol in post-menopausal women [52]. Molecular modeling revealed that carnosol fits within the ligand binding domain of both estrogen receptors (ERα and ERβ) and androgen receptor (AR) [53], and further studies using a time-resolved fluorescence resonance energy transfer assay found that carnosol interacts with both ER and AR receptors as an antagonist [53]. In prostate (LNCaP, 22Rv1) and breast cancer (MCF7) cells, carnosol (20–40 µM) decreased ER and AR receptor levels. Administration of carnosol in mice xenografted with prostate cancer cells (22Rv1) significantly reduced tumor growth. These data indicate potential anti-estrogen and anti-androgen properties of carnosol [53]. Both estrogen receptors (ERα and ERβ) were found to be expressed in muscle [54]. Estrogen stimulates glucose uptake in muscle cells and, importantly, the GLUT4 glucose transporter expression was significantly reduced in estrogen receptor ERα (−/−) mice [54], indicating a strong influence of estrogen receptor in muscle GLUT4 expression. In C_2_C_12_ mouse skeletal muscle cells, resveratrol (0.1 µM/L) increased glucose uptake and GLUT4 translocation and expression by a mechanism that involved ERα [55]. Based on these pieces of evidence and the finding that L6 cells express estrogen receptors [54], it is possible that the carnosol-stimulated glucose uptake, observed in the current study, involves ERα.

## 4. Materials and Methods

### 4.1. Materials

All cell culture materials, including antibiotic/antimycotic and trypsin solutions, were purchased from GIBCO Life Technologies (Burlington, ON, Canada). Insulin (Humulin R) was from Eli Lilly (Indianapolis, IN, USA). Total and phospho-specific Akt, AMPK, mTOR, ACC, LKB1, horseradish peroxidase (HRP)-conjugated anti-rabbit antibodies, and LumiGLOW reagents were from New England Biolabs (NEB) (Mississauga, ON, Canada). The anti-myc (9E10) and GLUT4 antibody was from Santa Cruz (Santa Cruz, CA, USA) and HRP-conjugated donkey anti-mouse immunoglobulin G (IgG) from Jackson ImmunoResearch Labs (West Grove, PA, USA). The Bradford protein assay reagent, electrophoresis reagents, molecular weight protein standards, and polyvinylidene difluoride (PVDF) membranes were from BioRad (Hercules, CA, USA). [3H]-2-deoxy-d-glucose was from PerkinElmer (Boston, MA, USA). Dried rosemary (*Rosmarinus officinalis* L.) leaves were from Compliments/Sobeys (Mississauga, ON, Canada), and rosemary extract was prepared as previously described [21]. Compound C and bovine serum albumin were from Calbiochem (Gibbstown, NJ, USA). Dimethyl sulfoxide (DMSO), metformin, cytochalasin B, carnosol, STO-609, (5*Z*)-oxozeaenol (OZ), phenylmethylsulfonyl fluoride (PMSF), and o-phenylenediamine dihydrochloride (OPD) were from Sigma (Oakville, ON, Canada).

### 4.2. Cell Culture, Treatment, and Glucose Uptake Assay

L6 myotubes were used in all experiments. Parental and GLUT4myc overexpressing L6 rat muscle cells were grown and differentiated into myotubes, as previously described [2,16,17]. Prior to any treatment, the cells were serum-deprived for 3 h. All treatments were performed using serum-deprived media. After treatment, the cells were rinsed with HEPES-buffered saline (HBS) followed by incubation with 10 μM [3*H*]-2-deoxy-d-glucose in HBS for 10 min to determine glucose uptake, as previously established [16,17]. Cytochalasin B (10 μM) was used to assess the non-specific glucose uptake. At the end of the treatment, the cells were washed with cold 0.9% NaCl solution, lysed with 0.05 N NaOH, and radioactivity measured by liquid scintillation counting. The Bradford assay was used to determine cellular protein levels.

### 4.3. Immunoblotting

Following treatment, the cells were washed with ice cold HBS solution and then lysed with ice cold lysis buffer. Whole cell lysates were prepared and then stored in −20 °C. Protein samples (15 µg) were separated using denaturing conditions sodium dodecyl sulfate polyacrylamide gel electrophoresis (SDS-PAGE) followed by a transfer to a PVDF membrane. The membranes were blocked for 1 h with 5% (*w*/*v*) dry milk dissolved in Tris-buffered saline and incubated overnight at 4 °C with the primary antibody. HRP-conjugated anti-rabbit secondary antibody was used (1 h incubation at room temperature) followed by exposure to LumiGLOW reagent and visualization of the corresponding bands using FluroChem software (Thermo Fisher, Waltham, MA, USA). 

### 4.4. GLUT4myc Translocation Assay

GLUT4myc overexpressing myotubes were treated and then fixed with 3% paraformaldehyde for 10 min at 4 °C. The cells were then incubated with 1% glycine and blocked using 10% goat serum in PBS. Anti-myc antibody was added and followed by incubation with horseradish peroxidase-conjugated donkey anti-mouse IgG. The cells were washed and the OPD reagent was added for 30 min at room temperature. The OPD reagent is a water-soluble substrate for horseradish peroxidase (HRP) that generates a yellow-orange product detectable at 492 nm by an enzyme-linked immunosorbent assay (ELISA) plate reader. The changes in the color corresponds to the amount of GLUT4myc transporters present in the plasma membrane. The reaction was stopped using 3 N HCl. The supernatant was collected from the well and its absorbance measured at 492 nm.

### 4.5. Statistical Analysis

Analysis of variance (ANOVA) followed by Tukey’s post-hoc analysis was used to determine the significance of the differences between groups. GraphPad Prism (v.7) software (GraphPad Software Inc., La Jolla, CA, USA) was used for calculations. Statistical significant was assumed at *p* < 0.05 (*), *p* < 0.01 (**), or *p* < 0.001 (***).

## 5. Conclusions

In summary, our study shows that, in L6 muscle cells, treatment with carnosol leads to a significant increase in glucose uptake to levels similar to insulin and metformin. The glucose uptake was significantly inhibited in the presence of compound C, an inhibitor of AMPK, but was not affected by wortmannin, an inhibitor of PI3K, indicating a mechanism that is dependent on AMPK. Overall, more studies should be performed to further investigate the potential of carnosol to be used to prevent and/or manage insulin resistance and T2DM.
